# Association Between Traumatic Brain Injury and Cognitive Decline Among Middle-to-Older Aged Men in the Vietnam Era Twin Study of Aging

**DOI:** 10.1089/neur.2024.0034

**Published:** 2024-06-17

**Authors:** Alexander Ivan B. Posis, John E. Alcaraz, Humberto Parada, Aladdin H. Shadyab, Jeremy A. Elman, Matthew S. Panizzon, Chandra A. Reynolds, Carol E. Franz, William S. Kremen, Linda K. McEvoy

**Affiliations:** ^1^ Herbert Wertheim School of Public Health and Human Longevity Science, University of California San Diego, La Jolla, California, USA.; ^2^ School of Public Health, San Diego State University, San Diego, California, USA.; ^3^ Moores Cancer Center, UC San Diego Health, La Jolla, California, USA.; ^4^ Department of Radiation Medicine & Applied Science, University of California, San Diego, La Jolla, California, USA.; ^5^ Department of Psychiatry, University of California San Diego, La Jolla, California, USA.; ^6^ Center for Behavior Genetics of Aging, University of California San Diego, La Jolla, California, USA.; ^7^ Institute for Behavioral Genetics, University of Colorado Boulder, Boulder, Colorado, USA.; ^8^ Kaiser Permanente Washington Health Research Institute, Seattle, Washington, USA.

**Keywords:** aging, cognition, epidemiology, prospective cohort, traumatic brain injury

## Abstract

Traumatic brain injury (TBI) is associated with increased risk of dementia. However, whether TBI is associated with greater cognitive decline over time in specific cognitive domains among older adults is not well understood. This prospective cohort study used data from 1476 male Vietnam Era Twin Study of Aging participants (average age at study entry = 57.9 years, range = 51–71 years; 97.6% non-Hispanic; 92.5% White) collected from 2003 to 2019, who had complete information on prior TBI. Participants completed a comprehensive neuropsychological assessment at up to three visits over up to a 12-year follow-up period during which they also self-reported their history of TBI. Multivariable, linear mixed-effects models were used to assess associations between TBI and cognitive performance trajectories. Effect measure modification by apolipoprotein E (*APOE*) epsilon 4 (*ε4*) genotype status was assessed in a subset of participants. Thirty-one percent of participants reported a history of TBI; 29.4% were *APOE* *ε4* carriers. There were no statistically significant associations of TBI with decline in episodic memory, executive function, or processing speed among participants overall. In models stratified by *APOE* *ε4* carrier status, TBI was associated with a larger magnitude of decline in executive function for *APOE* *ε4* carriers (β = −0.0181; 95% confidence interval [CI] −0.0335, −0.0027) compared to noncarriers (β = −0.0031; 95% CI −0.0128, 0.0067; *P*_Interaction_ = 0.03). In sensitivity analyses, TBI earlier in life (before military induction, average age = 20 years) was associated with faster declines in executive function compared to no TBI, irrespective of *APOE* *ε4* status. In this sample of middle-to-older aged men, TBI was associated with faster declines in executive function among *APOE* *ε4* carriers and among those who reported TBI in early life. These findings support the importance of a life course perspective when considering factors that may influence cognitive health in aging.

## Introduction

Traumatic brain injury (TBI) is a leading cause of death and disability^1^ and is associated with 84% higher risk of all-cause dementia^2^ and with earlier dementia and cognitive impairment onset.^3–5^ However, associations between TBI and cognitive test performance are mixed.^6–15^ Two recent prospective studies showed conflicting results.^14,15^ Among 8,662 men from the National Academy of Sciences-National Research Center (NAS-NRC) Twin Registry of World War II Veterans (mean age = 67 years), TBI was associated with greater 12-year declines in a global test of cognitive status, the Telephone Interview for Cognitive Status–modified (TICS-m), but specific cognitive domains were not assessed.^14^ In a study of 15,764 participants from the PROTECT-TBI cohort study (mean age = 62.7 years), TBI was associated with lower baseline measures of executive function, working memory, episodic memory, and processing speed but not with the rate of decline over up to 4 years of follow-up.^15^ These results were similar to those reported by a study of 53 National Football League retirees aged ≥50 years, compared to 26 controls and 83 clinical patients, that found baseline but not longitudinal associations between head injury and cognitive performance.^16^ Studies with more extensive follow-up and assessment of different cognitive domains are needed to better understand associations of TBI with age-related cognitive decline.

It is also unclear whether the apolipoprotein E (*APOE*) epsilon 4 (*ε4*) polymorphism, which confers greater genetic susceptibility for late-onset Alzheimer’s disease,^17^ is associated with greater TBI-related cognitive decline. A meta-analysis concluded that the ε4 allele confers a small risk for worse post-TBI outcomes but noted conflicting studies.^18^ It is possible that *APOE* *ε4* effects may differ by cognitive domain.

Therefore, the objective of this study was to leverage the detailed neuropsychological data assessed repeatedly over a long follow-up period (up to 12 years) in the Vietnam Era Twin Study of Aging (VETSA) to test the associations between lifetime TBI and later-life cognitive trajectories. Although prior cross-sectional work in VETSA found no main effect of TBI on episodic memory, executive function, and processing speed,^13^ longitudinal trajectories of cognitive performance as a function of TBI status, and differences by *APOE* *ε4* carrier status, have not been examined. We hypothesized that TBI would be associated with faster declines in episodic memory, executive function, and processing speed, and these declines would be greater among TBI participants who were *APOE* *ε4* carriers compared to noncarriers.

## Methods

### Study population

This prospective cohort study used data from VETSA, an ongoing longitudinal study of male twin pairs.^19,20^ The study design is described in detail elsewhere.^19,20^ Briefly, VETSA includes 1608 men who were recruited from the Vietnam Era Twin Registry (VETR), a nationally representative registry, which includes male twins, who were in the military at some time during the Vietnam era (1965–1975). VETSA participants were recruited at random from the 1992 Harvard Twin Study of Substance Abuse among VETR twins, a study that did not select participants on the basis of any substance use or other characteristics.^21^ Because VETSA was designed to assess cognitive changes during the critical transition period from middle-age to early older age, recruitment into wave 1 was restricted to a narrow age range of 51–60 years, and participants were followed at planned intervals (wave 2 occurred approximately 6 years after wave 1; wave 3 occurred an average of 12 years after wave 1). VETSA was also designed to address biases due to attrition and practice effects by enrolling new participants from the VETR at waves 2 and 3 who were of similar age to VETSA participants.^22^ A total of 1237 men (age range = 51–61 years) were enrolled at wave 1 from 2003 to 2007. At wave 2 (2009–2013), a total of 247 new participants (age range = 55–67 years) were enrolled, and at wave 3 (2016–2019), 124 new participants were enrolled (age range = 63–71 years). This resulted in 1608 participants. At the time of enrollment, VETSA participants had similar health and lifestyle characteristics compared to U.S. men in their age cohort.^23^ Approximately, 80% of VETSA participants did not report combat exposure.^24^

Of the 1608 VETSA participants, we excluded 131 with incomplete data on lifetime TBI and 1 missing cognitive outcome data, yielding a final analytic sample of 1476 participants. Baseline was defined as the participant’s time of enrollment into VETSA, regardless of wave of enrollment.

Data collection occurred at two sites (San Diego, CA, and Boston, MA) using identical protocols. This study was approved by the University of California, San Diego, and Boston University Institutional Review Boards. All participants provided written informed consent.

### Outcomes: Cognitive test performance

We assessed the following three primary cognitive domains based on prior literature: (1) episodic memory, (2) executive function, and (3) processing speed.^6–15^ VETSA participants were administered a comprehensive neurocognitive battery at each wave, with multiple tests assessing each cognitive domain. Using genetically informed multivariate analyses, latent constructs for the domains of episodic memory, executive function, and processing speed were created.^25–28^ In secondary analyses, we examined associations of TBI with working memory (a subcomponent of executive function),^27^ verbal fluency,^29^ and semantic fluency (a subcomponent of verbal fluency).^29^ Methods for all outcomes are summarized in Supplementary Data S1.

Domain-specific factor scores, corrected for effects of practice and selective attrition, were derived for each participant at all 3 waves and standardized based on the means and standard deviations of scores at wave 1, as described previously.^22,30^

### Main exposure: TBI

Lifetime TBI was defined based on methods described previously.^13^ Briefly, participants were asked the following four questions regarding prior head injury at each wave: (1) “Have you ever had a severe head injury that was associated with loss of consciousness or confusion?” (2) “Did that head injury (any of those head injuries) result in your staying overnight in the hospital?” (3) “Have you ever been told by a doctor that you had a concussion (If “Yes,” how many times?)?” (4) “Altogether, how many different head injuries or concussions (all total) have you had?” Starting in wave 2, participants who reported head injury based on the four questions were asked details of each injury, including age, cause, receipt of medical attention or hospitalization, and presence/duration of loss of consciousness (LOC) and post-traumatic amnesia (PTA). TBI severity was defined as follows: mild as LOC ≤20 minutes and/or PTA <24 hours, moderate as LOC >20 minutes and/or PTA ≥24 hours to 7 days, and severe as LOC >1 day and/or PTA >7 days. History of lifetime symptomatic TBI was defined here as a binary variable (any/none).

### Effect modifier: Apolipoprotein E genotype

We examined effect modification by *APOE* *ε4* carrier status among 1342 participants with *APOE* genotype. Genotyping methods in VETSA are described in detail elsewhere.^31^ Participants were classified as an *APOE* *ε4* carrier if they had ≥1 *ε4* allele (*n* = 394) and as noncarriers otherwise.

### Covariates

Covariate information was derived from medical interviews and questionnaires completed at each wave. Covariates included age at VETSA enrollment, race/ethnicity, years of education, young adult general cognitive ability as measured by the Armed Forces Qualification Test (AFQT), and annual family income. The AFQT is a 100-item multiple-choice test, administered to service members before military induction (at an average age of 20 years)^32,33^ and is correlated with standard IQ (*r* = 0.84).^34^ AFQT scores were recorded as percentiles based on military norms and then scaled and normalized. We considered several time-varying covariates as follows: standardized body mass index (BMI), smoking status, alcohol use, substance abuse, relationship status, participation in religious activities, number of close friends, loneliness,^35,36^ social isolation, and elevated psychiatric symptoms.^35,37^ Given the correlation between depressive and post-traumatic stress symptoms,^13^ we defined scores ≥16 on the Center for Epidemiological Studies Depression Scale (CES-D)^35^ at any wave, or ≥36 on the PTSD Checklist—Civilian version^37^ starting in wave 2, as elevated psychiatric symptoms. Social isolation was assessed by the question, “How many people do you know who you can trust and confide in?” as defined in a prior VETSA study (≥1 confidant; no confidants).^38^ Analyses with CES-D did not include loneliness as a separate covariate given its inclusion in the scale.

### Statistical analysis

Participant characteristics at baseline (i.e., VETSA study entry) were summarized by TBI status using median (minimum and maximum) or counts (percentages). Group differences were assessed using linear mixed-effects models for continuous variables or mixed-effects multinomial logistic regression models for categorical variables, with a random intercept accounting for the correlation between twin pairs.

We fit linear mixed-effects models to estimate associations of TBI status with longitudinal cognitive performance trajectories. Time from baseline was calculated as the difference in age from the participant’s first visit to subsequent visits, which reflects within-participant change.^39–41^ We used three successively adjusted models as follows: Model 1 included the fixed effects of TBI, time, and a TBI-by-time interaction term and adjusted for baseline age (centered at 57.86 years, average study entry age), race/ethnicity, education, annual family income, and AFQT; Model 2 additionally adjusted for time-varying health-related and social characteristics, which included BMI, smoking status, alcohol use, substance abuse, relationship status, participation in religious activities, number of close friends, social isolation, and elevated psychiatric symptoms; and Model 3 additionally adjusted for *APOE* *ε4* carrier status. All models included random intercepts and a family-relatedness random effect to adjust for the correlation between twin pairs. We plotted predicted values of Model 2 for all outcomes by TBI status over time.

To assess effect measure modification, we stratified Model 2 by *APOE* *ε4* carrier status. *P*-values for interaction (*P*_Interaction_) were calculated using likelihood ratio tests, comparing fully adjusted models with and without a 3-way interaction of *APOE* *ε4* carrier status-by-TBI-by-time.

In sensitivity analyses, we tested different indicators of TBI: highest severity (mild; moderate/severe), multiple TBI (0; 1; ≥2), and timing of TBI (none; before military induction; after military induction). As *ε2* and *ε4* alleles may confer opposing risk for Alzheimer’s disease,^42^ we excluded *ε2*/*ε4* participants from *APOE* *ε4* stratified analyses. We repeated the primary analysis with working memory, verbal fluency, and semantic fluency as secondary outcomes.

Statistical analyses were conducted in R (version 4.2.2; R Core Team, 2022). We used the lme4 package for linear mixed-effects analyses^43^ and the sjPlot package for visualization.^44^ Statistical tests were two-tailed, and results with *p*-values  <0.05 were considered significant.

## Results

### Participant characteristics at study entry

Of the 1476 men, 31.3% reported ≥1 lifetime TBI (median age of first TBI = 18 years). Of those with TBI, 65.8% were of mild severity, 36.4% reported ≥2 TBIs, and 56.9% reported TBI history before military induction (age range of first TBI before military induction: 2–22 years, after military induction: 18–69 years). At study entry, median age was 58 (range = 51.1–71.1) years (Table 1). Most were non-Hispanic/Latino (97.6%) and White (92.4%). Compared to no TBI history, those reporting TBI were more likely to have completed >12 years of education, felt lonely 1 day in the past week, had significant depressive symptom severity, and had symptoms of post-traumatic stress disorder (PTSD). Relative to participants with complete follow-up visit (*n* = 284), those who did not were less likely to report TBI, but these groups did not differ in age, race/ethnicity, AFQT, income, or *APOE* *ε4* status (Supplementary Table S1).

**Table 1.** Participant Characteristics at First Visit by Traumatic Brain Injury Status

Characteristics	TBI status		*p*-Value
	No (*n* = 1014)	Yes (*n* = 462)	
Age			
Median (min, max)	57.9 (51.1, 71.1)	58.1 (51.1, 70.9)	0.17
Ethnicity			
Non-Hispanic or Non-Latino	983 (96.9%)	457 (98.9%)	0.73
Race			
White	930 (91.7%)	435 (94.2%)	
Education years			
≤12	423 (41.7%)	159 (34.4%)	0.03
13–14	283 (27.9%)	137 (29.7%)	
15–16	220 (21.7%)	122 (26.4%)	
>16	88 (8.7%)	44 (9.5%)	
AFQT			
Median (Min, Max)	0.270 (−1.29, 3.50)	0.300 (−1.13, 2.32)	0.20
Income			
<$40,000	145 (17.9%)	65 (19.9%)	0.46
$40,000–89,999	432 (53.3%)	168 (51.4%)	
≥$90,000	233 (28.8%)	94 (28.7%)	
BMI			
Median (Min, Max)	28.8 (17.8, 48.9)	29.3 (19.5, 54.7)	0.17
Smoking status			
Never	332 (32.9%)	170 (36.8%)	0.12
Former	473 (46.9%)	200 (43.3%)	
Current	204 (20.2%)	92 (19.9%)	
Alcohol use			
Never	62 (6.1%)	24 (5.2%)	0.69
Former	299 (29.6%)	140 (30.4%)	
Light	416 (41.2%)	175 (38.0%)	
Moderate	91 (9.0%)	58 (12.6%)	
Heavy	141 (14.0%)	64 (13.9%)	
Relationship status			
Married or in a relationship	797 (78.9%)	356 (77.4%)	0.61
Participation in religious activities			
≥3 times a month	462 (45.8%)	184 (40.1%)	0.11
Close friends			
≥3	930 (92.9%)	424 (92.6%)	0.14
Loneliness			
≥ 1 day in past week	220 (21.8%)	130 (28.3%)	0.01
Social isolation			
No confidants	27 (2.7%)	18 (3.9%)	0.76
Alcohol or drug abuse			
Yes	45 (4.4%)	27 (5.8%)	0.76
Depressive symptoms			
Yes	129 (13.3%)	83 (18.6%)	0.03
PTSD symptoms			
Yes	106 (12.4%)	81 (18.2%)	<0.01
*APOEε4* carrier			
Yes	290 (30.8%)	104 (25.9%)	0.31

*p*-Values were derived from linear mixed-effects models for continuous variables and mixed-effects multinomial logistic regression models for categorical variables. Models included a random intercept to adjust for the correlation between twin pairs. n missing: income = 339 (23.0%), BMI = 3 (0.2%), smoking status = 5 (0.3%), alcohol use = 6 (0.4%), relationship status = 6 (0.4%), participation in religious activities = 9 (0.6%), loneliness = 8 (0.5%), social isolation = 12 (0.8%), close friends = 17 (1.2%), alcohol or drug abuse = 1 (0.1%), AFQT = 20 (1.4%), depressive symptoms = 63 (4.3%), PTSD symptoms = 172 (11.7%), *APOE* *ε4* carrier = 134 (9.1%).

AFQT, Armed Forces Qualification Test; *APOE* ε4, apolipoprotein E epsilon 4;  BMI, body mass index; PTSD, post-traumatic stress disorder; TBI, traumatic brain injury; VETSA, Vietnam Era Twin Study of Aging.

### TBI status and cognitive function by time

**FIG. 1.**
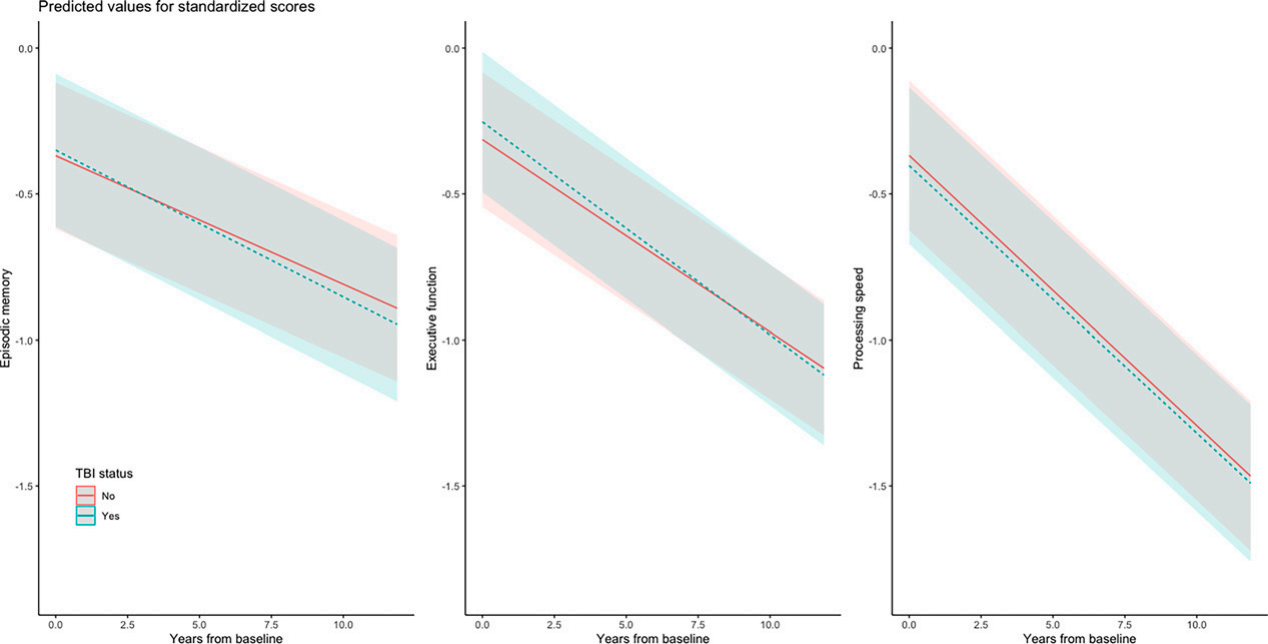
Trajectories of cognition function test performance over years from baseline by TBI. Plots are based on linear mixed-effects models adjusted for baseline age (centered at 57.86 years, the average age of entry into VETSA), race/ethnicity, education, annual family income, and young adult cognitive ability (AFQT at age 20), as well as time-varying BMI (standardized), smoking status, alcohol use, substance abuse, relationship status, participation in religious activities, number of close friends, social isolation, and elevated psychiatric symptoms. AFQT, Armed Forces Qualification Test; BMI, body mass index; TBI, traumatic brain injury; VETSA, Vietnam Era Twin Study of Aging.

Performance on episodic memory, executive function, and processing speed significantly declined over the 12-year follow-up period (βs for Time < −0.10; Table 2). Neither the main effect of TBI nor the TBI-by-time interaction was statistically significant in any model (Table 2; Fig. 1).

**Table 2.** Association of Any Traumatic Brain Injury with Cognitive Performance Trajectories over a 12-Year Follow-Up (*n* = 1476)

Outcome	Model	Term	β (95% CI)
Episodic memory	1	TBI	0.0045 (−0.1056; 0.1145)
		Time	−0.0455 (−0.0502; −0.0408)
		TBI by time	−0.0041 (−0.0126; 0.0045)
	2	TBI	0.019 (−0.0927; 0.1307)
		Time	−0.0441 (−0.0491; −0.039)
		TBI by time	−0.0063 (−0.0152; 0.0026)
	3	TBI	0.0187 (−0.0932; 0.1305)
		Time	−0.0438 (−0.0488; −0.0387)
		TBI by time	−0.0065 (−0.0154; 0.0024)
Executive function	1	TBI	0.0428 (−0.0592; 0.1449)
		Time	−0.067 (−0.0715; −0.0625)
		TBI by time	−0.0064 (−0.0145; 0.0017)
	2	TBI	0.0607 (−0.0429; 0.1644)
		Time	−0.0659 (−0.0705; −0.0612)
		TBI by time	−0.0071 (−0.0154; 0.0012)
	3	TBI	0.0627 (−0.0408; 0.1663)
		Time	−0.0656 (−0.0702; −0.0609)
		TBI by time	−0.0074 (−0.0156; 9e-04)
Processing speed	1	TBI	−0.0547 (−0.1721; 0.0628)
		Time	−0.0946 (−0.0992; −0.0899)
		TBI by time	0.0031 (−0.0054; 0.0116)
	2	TBI	−0.035 (−0.1502; 0.0802)
		Time	−0.0925 (−0.0975; −0.0875)
		TBI by time	9e-04 (−0.008; 0.0098)
	3	TBI	−0.0402 (−0.1558; 0.0753)
		Time	−0.0929 (−0.0979; −0.0879)
		TBI by time	0.0014 (−0.0075; 0.0103)

Beta (β) and 95% confidence intervals (CI) are derived from linear mixed-effects models that included random intercepts and family-relatedness as a random effect to adjust for correlation between twin pairs. Time is defined as years from baseline. Model 1 included the fixed effects of TBI, time, and a TBI-by-time interaction term, and adjusted for baseline age (centered at 57.86 years, the average age of entry into VETSA), race/ethnicity, education, annual family income, and young adult cognitive ability (AFQT at age 20). Model 2 additionally adjusted for time-varying BMI (standardized), smoking status, alcohol use, substance abuse, relationship status, participation in religious activities, number of close friends, social isolation, and elevated psychiatric symptoms. Model 3 additionally adjusted for *APOE* *ε4* carrier status among the subset of participants with *APOE* genotype data (*n* = 1342).

AFQT, Armed Forces Qualification Test; *APOE* ε4, apolipoprotein E epsilon 4; BMI, body mass index; TBI, traumatic brain injury; VETSA, Vietnam Era Twin Study of Aging.

### Effect measure modification by *APOE ε4* carrier status

**FIG. 2.**
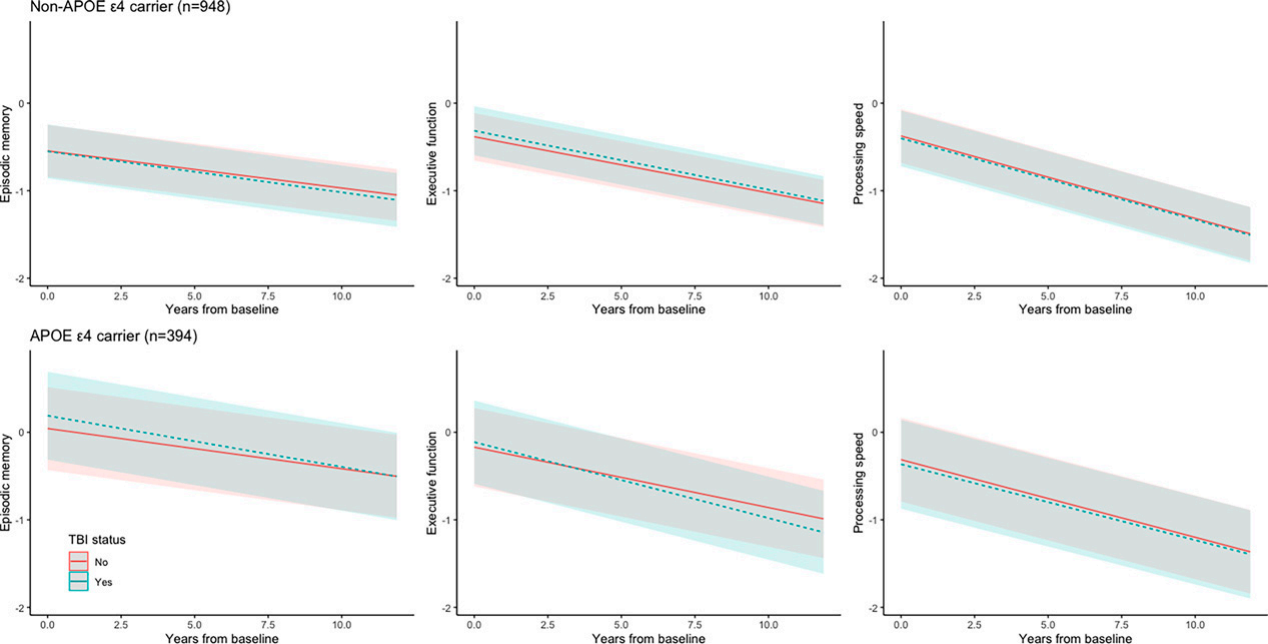
Trajectories of cognition function test performance over years from baseline by TBI stratified by *APOE* *ε4* carrier status. Plots are based on linear mixed-effects models adjusted for baseline age (centered at 57.86 years, the average age of entry into VETSA), race/ethnicity, education, annual family income, and young adult cognitive ability (AFQT at age 20), as well as time-varying BMI (standardized), smoking status, alcohol use, substance abuse, relationship status, participation in religious activities, number of close friends, social isolation, and elevated psychiatric symptoms. AFQT, Armed Forces Qualification Test; *APOE*, apolipoprotein E; BMI, body mass index; *ε4*, epsilon 4; TBI, traumatic brain injury; VETSA, Vietnam Era Twin Study of Aging.

Among the 1342 participants with *APOE* genotype data, 29.4% were *APOE* *ε4* carriers. Approximately, 25.9% of those with TBI were *APOE* *ε4* carriers compared to 30.8% of those without TBI; this difference was not statistically significant (*p* = 0.31). After stratification by *APOE* *ε4* carrier status, TBI was associated with greater decline in executive function for *APOE* *ε4* carriers (β = −0.0181; 95% confidence interval [CI] −0.0335, −0.0027) compared to noncarriers (β = −0.0031; 95% CI −0.0128, 0.0067; *P*_Interaction_ = 0.03; Table 3; Fig. 2). In other words, TBI was associated with a 0.18 standard deviation (SD) decline in executive function per decade among *APOE* *ε4* carriers.

**Table 3.** Association of Any Traumatic Brain Injury with Cognitive Performance Trajectories by *APOE* *ε4* Carrier Status over a 12-Year Follow-Up

			*APOEε4* carrier status		
			No (*n* = 948)	Yes (*n* = 394)	
Outcome	Model	Term	β (95% CI)	β (95% CI)	*P*_Interaction_
Episodic memory	1	TBI	−0.027 (−0.1573; 0.1034)	0.1421 (−0.0600; 0.3441)	0.49
		Time	−0.0442 (−0.0499; −0.0385)	−0.0474 (−0.0559; −0.0389)	
		TBI by time	−0.0021 (−0.0124; 0.0082)	−0.0103 (−0.0258; 0.0052)	
	2	TBI	−0.0047 (−0.1371; 0.1277)	0.1451 (−0.0615; 0.3516)	
		Time	−0.0427 (−0.0487; −0.0366)	−0.0459 (−0.0551; −0.0367)	
		TBI by time	−0.0041 (−0.0147; 0.0065)	−0.0124 (−0.0289; 0.0041)	
Executive function	1	TBI	0.0547 (−0.0654; 0.1748)	0.0494 (−0.1458; 0.2446)	0.03
		Time	−0.065 (−0.0704; −0.0597)	−0.0706 (−0.0786; −0.0627)	
		TBI by time	−0.0026 (−0.0123; 0.007)	−0.0169 (−0.0314; −0.0023)	
	2	TBI	0.0679 (−0.0531; 0.1889)	0.0612 (−0.1429; 0.2653)	
		Time	−0.0641 (−0.0697; −0.0586)	−0.0692 (−0.0779; −0.0606)	
		TBI by time	−0.0031 (−0.0128; 0.0067)	−0.0181 (−0.0335; −0.0027)	
Processing speed	1	TBI	−0.0537 (−0.1919; 0.0845)	−0.0336 (−0.255; 0.1879)	0.70
		Time	−0.0964 (−0.1021; −0.0907)	−0.0913 (−0.0996; −0.0829)	
		TBI by time	0.0031 (−0.0071; 0.0133)	0.0036 (−0.0116; 0.0187)	
	2	TBI	−0.0312 (−0.1697; 0.1074)	−0.0399 (−0.254; 0.1742)	
		Time	−0.0945 (−0.1005; −0.0884)	−0.0897 (−0.0987; −0.0807)	
		TBI by time	0.0018 (−0.0089; 0.0125)	0.0011 (−0.0149; 0.0170)	

Beta (β) and 95% confidence intervals (CI) are derived from linear mixed-effects models that included random intercepts and family-relatedness as a random effect to adjust for correlation between twin pairs. Time is defined as years from baseline. Model 1 included the fixed effects of TBI, time, and a TBI-by-time interaction term, and adjusted for baseline age (centered at 57.86 years, the average age of entry into VETSA), race/ethnicity, education, annual family income, and young adult cognitive ability (AFQT at age 20). Model 2 additionally adjusted for time-varying BMI (standardized), smoking status, alcohol use, substance abuse, relationship status, participation in religious activities, number of close friends, social isolation, and elevated psychiatric symptoms. *p*-Values for interaction (*P*_Interaction_) were calculated using likelihood ratio tests to compare fully adjusted models with and without a 3-way interaction of *APOE ε4* carrier status-by-TBI-by-time.

AFQT, Armed Forces Qualification Test; *APOE ε4*, apolipoprotein E epsilon 4; BMI, body mass index; TBI, traumatic brain injury; VETSA, Vietnam Era Twin Study of Aging.

### Sensitivity analysis

In sensitivity analyses, neither mild nor moderate/severe TBI was associated with the rate of decline for any cognitive domain (Supplementary Table S2). Further stratification by *APOE* *ε4* status showed that mild TBI was associated with greater decline in executive function among *APOE* *ε4* carriers, relative to no TBI (β = −0.0294; 95% CI −0.0474, −0.01; *P*_Interaction_ <0.01; Supplementary Table S3). TBI before military induction was associated with greater executive function decline compared to no TBI (β = −0.0104; 95% CI −0.0208, −1e-04; Supplementary Table S4); this was not modified by *APOE* *ε4* status (*P*_Interaction_ >0.10, all; Supplementary Fig. S1). TBI after military induction was not associated with decline in any domain, regardless of *APOE* *ε4* status. Associations did not differ by number of TBIs (Supplementary Table S5). Excluding *ε2*/*ε4* participants did not materially affect results (Supplementary Table S6). TBI was not associated with decline in any secondary cognitive outcome (Supplementary Table S7; Supplementary Fig. S2); this did not differ by *APOE* *ε4* status (*P*_Interaction_ > 0.10, all; Supplementary Table S8; Supplementary Fig. S3).

## Discussion

In this prospective cohort study of middle-to-older aged men, TBI was associated with significantly steeper declines in executive function, by 0.18 SD per decade, among *APOE* *ε4* carriers. Greater TBI-related decline in executive function was observed primarily among those reporting TBI earlier in life, before military induction. TBI was not associated with change in episodic memory or processing speed over a 12-year follow-up period. These findings contribute to our understanding of the role of TBI with cognitive health in aging, including the impact of *APOE* *ε4* and timing of injury.

Overall, we found that TBI was not associated with rate of decline in episodic memory, executive function, or processing speed, consistent with results from prior studies with shorter follow-up periods.^11,15^ However, we found that among *APOE* *ε4* carriers, TBI was associated with faster decline in executive function relative to those without TBI. This aligns with conclusions of a meta-analysis that found that the *APOE* *ε4* allele may be associated with worse long-term neurocognitive recovery from TBI.^18^ Our results differ from the NAS-NRC Twin Registry study, which did not find significant differences in the rate of global cognitive decline by *APOE*.^14^ However, the NAS-NRC study did not examine executive function, and our results suggest that the *APOE* *ε4* differences may be specific to this ability. In prior work with the VETSA sample, we found that *APOE* *ε4* was associated with a thinner frontal cortex,^45^ a vital structure for executive function. We also found that the *ε4* genotype conferred greater vulnerability to other adverse exposures, including higher vascular burden^46^ and lower testosterone.^47^ Taken together, these results suggest that the presence of an *APOE* *ε4* allele may increase vulnerability to adverse consequences of TBI on executive function, potentially through neurodegenerative or vascular mechanisms, which warrants further study.

In sensitivity analyses, we observed that TBI before military induction (mean age 20 years) was associated with greater executive function decline, relative to no TBI. This suggests that TBIs earlier in life may have stronger associations with later-life cognition than TBIs that occur later in life. It is plausible that the stronger effect of TBIs during this period may be due to interference with neurodevelopment, but additional research is needed. Conflicting results were reported in the NAS-NRC Twin Registry study, which found that TBI after age 25 (vs. before age 25) had stronger associations with TICS-m scores.^14^ Differences may be due to the older age of this World War II veteran cohort compared to VETSA and to the lack of executive function measures in the TICS-m. We did not find that those with multiple TBIs had faster cognitive decline. This may be due to the mild nature of most TBIs in our cohort and few participants reporting multiple TBIs.

Our study has several limitations. First, TBI was self-reported, potentially underestimating the prevalence of lifetime TBI. However, TBI information was derived from detailed items regarding the nature and plausibility of TBIs. Second, although there is potential for selection bias due to loss to follow-up, there were few differences between participants with complete and incomplete follow-up. Third, although we controlled for many confounding factors, there is always potential unmeasured or residual confounding. Lastly, this cohort comprised male veterans who were primarily non-Hispanic and White, reducing generalizability to other populations. However, participants had similar characteristics to American men in their age cohort,^23^ and most reported TBIs were before military induction and were not military-related.

This study had several strengths, which included longitudinal follow-up over a 12-year period. We assessed cognitive change using a rigorous neuropsychological battery and controlled for practice effects and effects of selective attrition.^22^ Our measures comprised multiple tests in each domain rather than single brief tests, which provides substantial increases in sensitivity.^26,48^ We were able to control for time-varying covariates, and young adult cognitive ability, which is a predictor of later-life cognitive function and allows for improved assessment of domain-specific decline.^49^

In this prospective cohort study of middle-to-older aged male veterans, TBI history was associated with a small but significant increase in the rate of executive function decline among *APOE* *ε4* carriers and among those who reported TBI earlier in life, before military service. This emphasizes the importance of preventing earlier life TBIs, which occur during periods of substantial brain development. Given that our cohort is younger than the age at which age-related cognitive decline substantially accelerates, the small magnitudes of association observed here may be more pronounced with further follow-up at later ages in this cohort. More research is needed to assess other life course factors, alongside TBI and *APOE* *ε4*, that affect later-life cognitive decline. In addition, further research is needed among more racially and ethnically diverse populations, and populations including women and older adults, to more fully understand who is at greatest risk for cognitive impairment related to TBI and to identify the cognitive domains that are most vulnerable to TBI.

## Supplementary Material

Supplementary Data S1

## Supplementary Material

Supplementary Figure S1

## Supplementary Material

Supplementary Figure S2

## Supplementary Material

Supplementary Figure S3

## Supplementary Material

Supplementary Table S1

## Supplementary Material

Supplementary Table S2

## Supplementary Material

Supplementary Table S3

## Supplementary Material

Supplementary Table S4

## Supplementary Material

Supplementary Table S5

## Supplementary Material

Supplementary Table S6

## Supplementary Material

Supplementary Table S7

## Supplementary Material

Supplementary Table S8

## Abbreviations Used

AFQT: Armed Forces Qualification Test
APOE ε4: apolipoprotein E epsilon 4
BMI: body mass index
CA: California
CES-D: Center for Epidemiologic Studies Depression Scale
CI: confidence interval
IQ: intelligence quotient
LOC: loss of consciousness
MA: Massachusetts
NAS-NRC: National Academy of Sciences-National Research Center
PInteraction: P values for interaction
PTA: post-traumatic amnesia
PTSD: post-traumatic stress disorder
SD: standard deviation
TBI: traumatic brain injury
TICS-m: Telephone Interview for Cognitive Status-modified
VETR: Vietnam Era Twin Registry
VETSA: Vietnam Era Twin Study of Aging
