# Supplementary material for: Association Between Traumatic Brain Injury and Cognitive Decline Among Middle-to-Older Aged Men in the Vietnam Era Twin Study of Aging
Source: Neurotrauma Rep. 2024 Jun 17;5(1):563–73. doi: 10.1089/neur.2024.0034 (PMC11257108; doi:10.1089/neur.2024.0034)
Supplement: Supplementary Data S1 [file neur.2024.0034_supplementarymethods1.docx]

**Supplementary Methods 1**: Description of cognitive outcome methods

VETSA participants were administered a comprehensive neurocognitive battery at each wave, with multiple tests assessing multiple cognitive domains of interest. Latent constructs for the domains of episodic memory,^1,2^ executive function,^3^ processing speed,^4^ working memory,^3^ verbal fluency,^5^ and semantic fluency^5^ were created using genetically-informed multivariate analyses. For the present study, we focused on 3 outcomes: 1) episodic memory, executive function, and processing speed. We also included 3 secondary outcomes: 1) working memory, 2) verbal fluency, and 3) semantic fluency.

Episodic memory was derived using the California Verbal Learning Test-2 (CVLT-II) short and long delay free recall,^6^ the Wechsler Memory Scale (WMS)-III Logical Memory immediate and delayed recall,^7^ and the Visual Reproductions tests immediate and delayed recall.^1,2^

Executive function was derived from multiple tests as follows: Number correct on color-word test, adjusted for performance on color naming and word reading tests from the Stroop Test,^8^ letter-number sequencing and digit span tests from the WMS-III,^7^ reading span task,^9^ and letter-number switching time adjusted for time on letter and number sequencing tasks from the Delis-Kaplan Executive Function System (D-KEFS) Trail Making Test, and category-switching score after adjusting for category fluency score on the D-KEFS Category switching tests.^3,10^ The working memory factor explains the variance related to working memory span that was not captured by the executive factor score.^3^ While the working memory and executive function scores are correlated, we analyzed them separately to allow for comparability with other studies.

Processing speed was derived from scores on the number and letter sequencing task of the D-KEFS Trail-Making Test,^10^ color naming and word reading subtests of the Stroop Test,^8^ and the Simple Reaction Time task.^4^

Verbal fluency was derived from Letter fluency (F, A, and S), semantic fluency (Animals and Boys’ Names) category switching subtest (fruits and items of furniture) from the D-KEFS.^5^ The semantic fluency factor explains the variance related to category fluency that was not captured by general verbal fluency factor score.^5^

*References for Supplementary Methods 1:*

1. Kremen WS, Panizzon MS, Franz CE, et al. Genetic complexity of episodic memory: a twin approach to studies of aging. Psychol Aging. 2014;29(2):404-417. doi:10.1037/a0035962
2. Gustavson DE, Elman JA, Sanderson‐Cimino M, et al. Extensive memory testing improves prediction of progression to MCI in late middle age. Alzheimers Dement (Amst). 2020;12(1):e12004. doi:10.1002/dad2.12004
3. Gustavson DE, Panizzon MS, Franz CE, et al. Genetic and environmental architecture of executive functions in midlife. Neuropsychology. 2018;32(1):18-30. doi:10.1037/neu0000389
4. Sanderson-Cimino M, Panizzon MS, Elman JA, et al. Genetic and environmental architecture of processing speed across midlife. Neuropsychology. 2019;33(6):862-871. doi:10.1037/neu0000551
5. Gustavson DE, Panizzon MS, Elman JA, et al. Genetic and environmental influences on verbal fluency in middle age: a longitudinal twin study. Behav Genet. 2018;48(5):361-373. doi:10.1007/s10519-018-9910-6
6. Delis DC, Kaplan E, Kramer JH, Ober BA. California Verbal Learning Test (CVLT). Psychological Corporation; 2000.
7. Wechsler D. Wechsler Memory Scale (WMS-III). Psychological Corporation; 1997.
8. Golden CJ, Freshwater SM. Stroop Color and Word Test: A Manual for Clinical and Experimental Uses.; 2002.
9. Daneman M, Carpenter PA. Individual differences in working memory and reading. Journal of Verbal Learning and Verbal Behavior. 1980;19(4):450-466. doi:10.1016/S0022-5371(80)90312-6
10. Delis DC, Kaplan E, Kramer JH. Delis-Kaplan Executive Function System (D-KEFS). Psychological Corporation; 2001.
