# Supplementary material for: Association Between Traumatic Brain Injury and Cognitive Decline Among Middle-to-Older Aged Men in the Vietnam Era Twin Study of Aging
Source: Neurotrauma Rep. 2024 Jun 17;5(1):563–73. doi: 10.1089/neur.2024.0034 (PMC11257108; doi:10.1089/neur.2024.0034)
Supplement: Supplementary Figure S1 [file neur.2024.0034_supplementaryfigure1.docx]

**
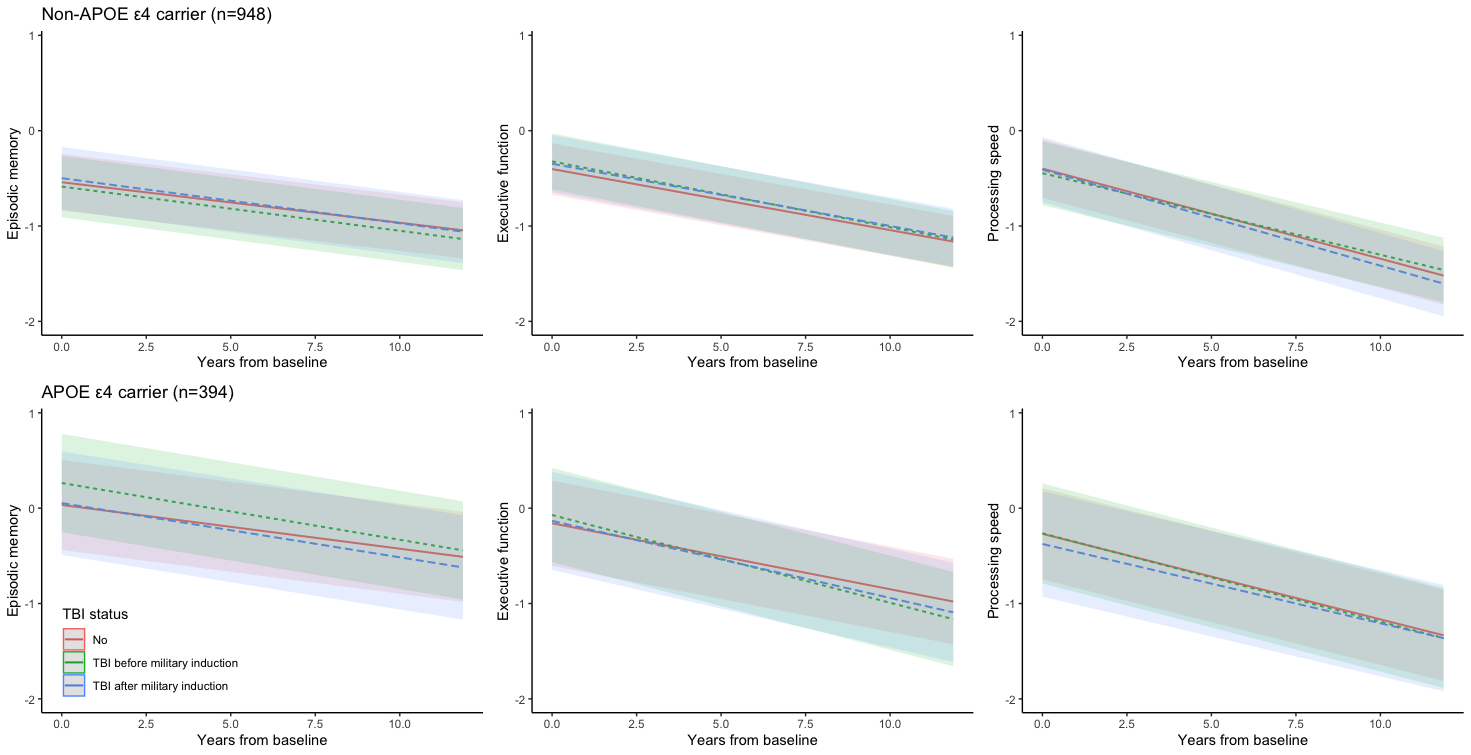
**

**Supplementary Figure 1.** Trajectories of cognition function test performance over years from baseline by TBI before or after military induction vs. no TBI, stratified by APOE ε4 carrier status. Plots are based on linear mixed-effects models adjusted for baseline age (centered at 57.86 years, the average age of entry into VETSA), race/ethnicity, education, annual family income and young adult cognitive ability (AFQT at age 20) as well as time-varying BMI (standardized), smoking status, alcohol use, substance abuse, relationship status, participation in religious activities, number of close friends, loneliness, social isolation, and elevated psychiatric symptoms.
