# Supplementary material for: Association Between Traumatic Brain Injury and Cognitive Decline Among Middle-to-Older Aged Men in the Vietnam Era Twin Study of Aging
Source: Neurotrauma Rep. 2024 Jun 17;5(1):563–73. doi: 10.1089/neur.2024.0034 (PMC11257108; doi:10.1089/neur.2024.0034)
Supplement: Supplementary Figure S2 [file neur.2024.0034_supplementaryfigure2.docx]

**
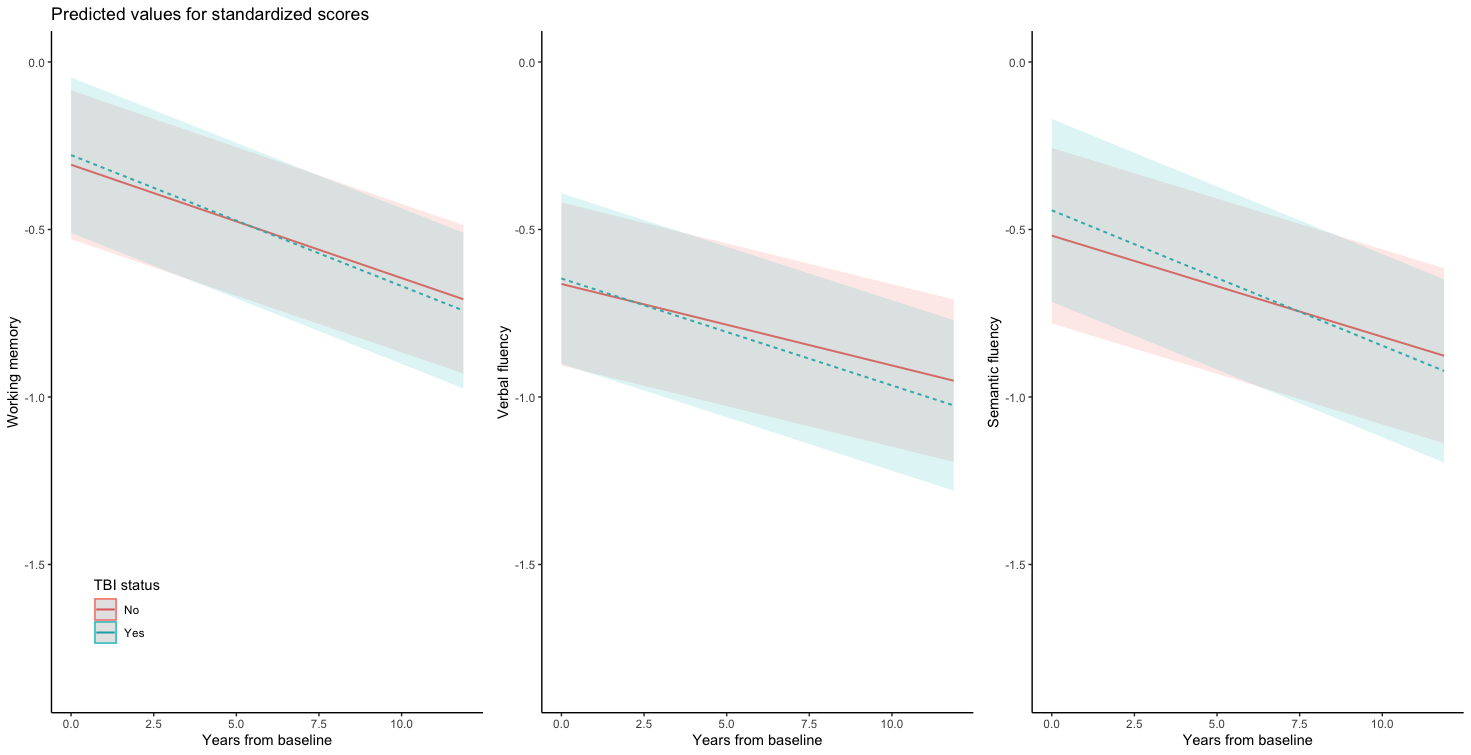
**

**Supplementary Figure 2.** Trajectories of secondary cognition function test performance outcomes over years from baseline by TBI. Plots are based on linear mixed-effects models adjusted for baseline age (centered at 57.86 years, the average age of entry into VETSA), race/ethnicity, education, annual family income and young adult cognitive ability (AFQT at age 20) as well as time-varying BMI (standardized), smoking status, alcohol use, substance abuse, relationship status, participation in religious activities, number of close friends, loneliness, social isolation, and elevated psychiatric symptoms.
