# Supplementary material for: Association Between Traumatic Brain Injury and Cognitive Decline Among Middle-to-Older Aged Men in the Vietnam Era Twin Study of Aging
Source: Neurotrauma Rep. 2024 Jun 17;5(1):563–73. doi: 10.1089/neur.2024.0034 (PMC11257108; doi:10.1089/neur.2024.0034)
Supplement: Supplementary Table S1 [file neur.2024.0034_supplementarytable1.docx]

| **Supplementary Table 1:** Participant characteristics by follow up status | | | | |
| --- | --- | --- | --- | --- |
|  |  | Followed for 2nd or 3rd visit | |  |
| Characteristic |  | No (N=284) | Yes (N=1192) | *p-*value |
| TBI | Yes | 72 (25.4%) | 390 (32.7%) | <0.01 |
| Age | Median [Min, Max] | 57.7 [51.8, 65.9] | 58.0 [51.1, 71.1] | 0.82 |
| Ethnicity | Non-Hispanic or Non-Latino | 276 (97.2%) | 1164 (97.7%) | 0.73 |
| Race | White | 265 (93.3%) | 1100 (92.3%) |  |
| Education years | ≤12 | 131 (46.1%) | 451 (37.8%) | 0.03 |
|  | 13-14 | 74 (26.1%) | 346 (29.0%) |  |
|  | 15-16 | 58 (20.4%) | 284 (23.8%) |  |
|  | >16 | 21 (7.4%) | 111 (9.3%) |  |
| AFQT | Median [Min, Max] | 0.250 [-1.13, 3.50] | 0.300 [-1.29, 2.32] | 0.51 |
| Income | <$40,000 | 46 (19.5%) | 164 (18.2%) | 0.52 |
|  | $40,000-$89,999 | 129 (54.7%) | 471 (52.3%) |  |
|  | ≥$90,000 | 61 (25.8%) | 266 (29.5%) |  |
| BMI | Median [Min, Max] | 29.4 [17.8, 48.7] | 28.9 [19.3, 54.7] | 0.48 |
| Smoking status | Never | 77 (27.1%) | 425 (35.8%) | 0.01 |
|  | Former | 118 (41.5%) | 555 (46.8%) |  |
|  | Current | 89 (31.3%) | 207 (17.4%) |  |
| Alcohol use | Never | 19 (6.7%) | 67 (5.6%) | 0.91 |
|  | Former | 96 (33.9%) | 343 (28.9%) |  |
|  | Light | 95 (33.6%) | 496 (41.8%) |  |
|  | Moderate | 25 (8.8%) | 124 (10.4%) |  |
|  | Heavy | 48 (17.0%) | 157 (13.2%) |  |
| Relationship status | Married or in a relationship | 214 (76.4%) | 939 (78.9%) | 0.32 |
| Participation in religious activities | ≥3 times a month | 114 (40.7%) | 532 (44.8%) | 0.22 |
| Close friends | ≥3 | 249 (89.9%) | 1105 (93.5%) | 0.50 |
| Loneliness | At least 1 day in the past week | 73 (26.0%) | 277 (23.3%) | 0.41 |
| Social isolation | No confidants | 10 (3.6%) | 35 (3.0%) | 0.57 |
| Alcohol or drug abuse | Yes | 19 (6.7%) | 53 (4.4%) | 0.82 |
| Depressive symptoms | Yes | 49 (17.9%) | 163 (14.3%) | * |
| APOE ε4 carrier | Yes | 92 (32.6%) | 302 (28.5%) | 0.14 |
| *P-*values were derived from linear mixed-effects models for continuous variables and mixed-effects multinomial logistic regression models for categorical variables. Models included a random intercept to adjust for the correlation between twin pairs. The test for race and ethnicity were combined given sparse cell counts. *No test was reported for depressive symptom severity due to model fit. | | | | |
| n missing: income = 339 (23.0%), BMI = 3 (0.2%), smoking status = 5 (0.3%), alcohol use = 6 (0.4%), relationship status = 6 (0.4%), participation in religious activities =9 (0.6%), loneliness = 8 (0.5%), social isolation = 12 (0.8%), close friends = 17 (1.2%), alcohol or drug abuse = 1 (0.1%), AFQT = 20 (1.4%), depressive symptoms = 63 (4.3%), APOE ε4 carrier = 134 (9.1%) | | | | |
