# Supplementary material for: Association Between Traumatic Brain Injury and Cognitive Decline Among Middle-to-Older Aged Men in the Vietnam Era Twin Study of Aging
Source: Neurotrauma Rep. 2024 Jun 17;5(1):563–73. doi: 10.1089/neur.2024.0034 (PMC11257108; doi:10.1089/neur.2024.0034)
Supplement: Supplementary Table S2 [file neur.2024.0034_supplementarytable2.docx]

| **Supplementary Table 2:** Association of traumatic brain injury by severity with cognitive performance trajectories over a 12 year of follow up | | | |
| --- | --- | --- | --- |
| Outcome | Term |  | β (95% CI) |
| Episodic memory | TBI (ref = no TBI) | Mild | -0.0029 (-0.1331; 0.1272) |
|  |  | Moderate/severe | 0.1191 (-0.0668; 0.3051) |
|  | Time |  | -0.0438 (-0.0488; -0.0388) |
|  | TBI by time (ref = no TBI) | Mild | -0.0089 (-0.0192; 0.0014) |
|  |  | Moderate/severe | -0.0086 (-0.0239; 0.0066) |
| Executive function | TBI (ref = no TBI) | Mild | 0.0641 (-0.0566; 0.1848) |
|  |  | Moderate/severe | 0.0667 (-0.1062; 0.2396) |
|  | Time |  | -0.0656 (-0.0702; -0.0609) |
|  | TBI by time (ref = no TBI) | Mild | -0.009 (-0.0186; 5e-04) |
|  |  | Moderate/severe | -0.0041 (-0.0183; 0.0101) |
| Processing speed | TBI (ref = no TBI) | Mild | 0.0275 (-0.087; 0.142) |
|  |  | Moderate/severe | 0.0442 (-0.119; 0.2073) |
|  | Time |  | -0.0337 (-0.038; -0.0295) |
|  | TBI by time (ref = no TBI) | Mild | -0.005 (-0.0137; 0.0037) |
|  |  | Moderate/severe | -0.0092 (-0.0221; 0.0036) |
| *Note*: Beta (β) and 95% confidence intervals (CI) are derived from linear mixed-effects models that included random intercepts and family-relatedness a random effect to adjust for correlation between twin pairs. Time is defined as years from baseline. Models include fixed effects of TBI, time, and a TBI by time interaction term, and are adjusted for baseline age (centered at 57.86 years, the average age of entry into VETSA), race/ethnicity, education, annual family income, young adult cognitive ability (AFQT at age 20) and APOE ε4 carrier status.as well as time-varying BMI (standardized), smoking status, alcohol use, substance abuse, relationship status, participation in religious activities, number of close friends, social isolation, and elevated psychiatric symptoms. | | | |
