# Supplementary material for: Association Between Traumatic Brain Injury and Cognitive Decline Among Middle-to-Older Aged Men in the Vietnam Era Twin Study of Aging
Source: Neurotrauma Rep. 2024 Jun 17;5(1):563–73. doi: 10.1089/neur.2024.0034 (PMC11257108; doi:10.1089/neur.2024.0034)
Supplement: Supplementary Table S3 [file neur.2024.0034_supplementarytable3.docx]

| **Supplementary Table 3:** Association of traumatic brain injury by severity with cognitive performance trajectories by among those with and without an APOE e4 allele | | | | | |
| --- | --- | --- | --- | --- | --- |
|  |  |  | APOE ε4 carrier status | |  |
|  |  |  | No (n=948) | Yes (n=394) |  |
| Outcome | Term |  | β (95% CI) | β (95% CI) | *P*_Interaction_ |
| Episodic memory | TBI (ref = no TBI) | Mild | -0.0288 (-0.1828; 0.1252) | 0.137 (-0.1043; 0.3783) | 0.585 |
|  |  | Moderate/severe | 0.0775 (-0.1426; 0.2976) | 0.3036 (-0.0419; 0.6491) |  |
|  | Time |  | -0.0427 (-0.0487; -0.0367) | -0.046 (-0.0553; -0.0368) |  |
|  | TBI by time (ref = no TBI) | Mild | -0.008 (-0.0203; 0.0042) | -0.0131 (-0.0323; 0.006) |  |
|  |  | Moderate/severe | -0.0024 (-0.0204; 0.0157) | -0.0188 (-0.0474; 0.0097) |  |
| Executive function | TBI (ref = no TBI) | Mild | 0.0559 (-0.0841; 0.1959) | 0.1045 (-0.1336; 0.3426) | 0.005 |
|  |  | Moderate/severe | 0.0767 (-0.1234; 0.2768) | 0.0349 (-0.3137; 0.3835) |  |
|  | Time |  | -0.0641 (-0.0696; -0.0586) | -0.0695 (-0.0781; -0.0608) |  |
|  | TBI by time (ref = no TBI) | Mild | -0.0012 (-0.0124; 0.0101) | -0.0294 (-0.0474; -0.0114) |  |
|  |  | Moderate/severe | -0.0106 (-0.0274; 0.0062) | 0.0126 (-0.0142; 0.0395) |  |
| Processing speed | TBI (ref = no TBI) | Mild | -0.0305 (-0.1891; 0.1282) | -0.0807 (-0.3259; 0.1645) | 0.728 |
|  |  | Moderate/severe | -0.1148 (-0.3417; 0.1121) | 0.074 (-0.2761; 0.4241) |  |
|  | Time |  | -0.0944 (-0.1004; -0.0883) | -0.0895 (-0.0984; -0.0805) |  |
|  | TBI by time (ref = no TBI) | Mild | 0.0052 (-0.0071; 0.0176) | 0.009 (-0.0096; 0.0276) |  |
|  |  | Moderate/severe | -0.0077 (-0.0261; 0.0107) | -0.0131 (-0.0408; 0.0145) |  |

*Note*: Beta (β) and 95% confidence intervals (CI) are derived from linear mixed-effects models that included random intercepts and family-relatedness a random effect to adjust for correlation between twin pairs. Time is defined as years from baseline. Models included fixed effects of TBI, time, and a TBI by time interaction term, and adjusted for baseline age (centered at 57.86 years, the average age of entry into VETSA), race/ethnicity, education, annual family income, and young adult cognitive ability (AFQT at age 20) as well as time-varying BMI (standardized), smoking status, alcohol use, substance abuse, varying relationship status, participation in religious activities, number of close friends, social isolation, elevated psychiatric symptoms, and APOE ε4 carrier status. *P* values for interaction (*P*_Interaction_) were calculated using likelihood ratio tests to compare fully adjusted models with and without a 3-way interaction of APOE ε4 carrier status by TBI by time.
