# Supplementary material for: Association Between Traumatic Brain Injury and Cognitive Decline Among Middle-to-Older Aged Men in the Vietnam Era Twin Study of Aging
Source: Neurotrauma Rep. 2024 Jun 17;5(1):563–73. doi: 10.1089/neur.2024.0034 (PMC11257108; doi:10.1089/neur.2024.0034)
Supplement: Supplementary Table S4 [file neur.2024.0034_supplementarytable4.docx]

| **Supplementary Table 4:** Association of traumatic brain injury before and after military induction with cognitive performance trajectories over a 12 year follow up | | | |
| --- | --- | --- | --- |
| Outcome | Term |  | β (95% CI) |
| Episodic memory | TBI (ref = no TBI) | Before military induction | 0.0369 (-0.1026; 0.1765) |
|  |  | After military induction | -0.0074 (-0.159; 0.1442) |
|  | Time |  | -0.0435 (-0.0486; -0.0385) |
|  | TBI by time (ref = no TBI) | Before military induction | -0.0069 (-0.0181; 0.0043) |
|  |  | After military induction | -0.0066 (-0.0186; 0.0054) |
| Executive function | TBI (ref = no TBI) | Before military induction | 0.0928 (-0.0364; 0.2219) |
|  |  | After military induction | 0.0287 (-0.1121; 0.1694) |
|  | Time |  | -0.0655 (-0.0701; -0.0608) |
|  | TBI by time (ref = no TBI) | Before military induction | -0.0104 (-0.0208; -1e-04) |
|  |  | After military induction | -0.0041 (-0.0152; 0.0071) |
| Processing speed | TBI (ref = no TBI) | Before military induction | -0.021 (-0.1648; 0.1228) |
|  |  | After military induction | -0.0605 (-0.2168; 0.0957) |
|  | Time |  | -0.0927 (-0.0977; -0.0877) |
|  | TBI by time (ref = no TBI) | Before military induction | 0.0048 (-0.0063; 0.016) |
|  |  | After military induction | -0.0037 (-0.0157; 0.0083) |
| *Note*: Beta (β) and 95% confidence intervals (CI) are derived from linear mixed-effects models that included random intercepts and family-relatedness a random effect to adjust for correlation between twin pairs. Time is defined as years from baseline. Models include fixed effects of TBI, time, and a TBI by time interaction term, and are adjusted for baseline age (centered at 57.86 years, the average age of entry into VETSA), race/ethnicity, education, annual family income, young adult cognitive ability (AFQT at age 20) and APOE ε4 carrier status as well as time-varying BMI (standardized), smoking status, alcohol use, substance abuse, relationship status, participation in religious activities, number of close friends, social isolation, and elevated psychiatric symptoms. | | | |
