# Supplementary material for: Association Between Traumatic Brain Injury and Cognitive Decline Among Middle-to-Older Aged Men in the Vietnam Era Twin Study of Aging
Source: Neurotrauma Rep. 2024 Jun 17;5(1):563–73. doi: 10.1089/neur.2024.0034 (PMC11257108; doi:10.1089/neur.2024.0034)
Supplement: Supplementary Table S5 [file neur.2024.0034_supplementarytable5.docx]

| **Supplementary Table 5:** Association of traumatic brain injury count with cognitive performance trajectories over a 12 year of follow up | | | |
| --- | --- | --- | --- |
| Outcome | Term |  | β (95% CI) |
| Episodic memory | TBI (ref = no TBI) | 1 TBI | -0.0572 (-0.1855; 0.0712) |
|  |  | ≥2 TBIs | 0.1753 (0.0021; 0.3485) |
|  | Time |  | -0.0437 (-0.0488; -0.0387) |
|  | TBI by time (ref = no TBI) | 1 TBI | -0.0054 (-0.0157; 0.0049) |
|  |  | ≥2 TBIs | -0.009 (-0.0228; 0.0048) |
| Executive function | TBI (ref = no TBI) | 1 TBI | 0.0206 (-0.0984; 0.1396) |
|  |  | ≥2 TBIs | 0.1479 (-0.0128; 0.3085) |
|  | Time |  | -0.0655 (-0.0702; -0.0609) |
|  | TBI by time (ref = no TBI) | 1 TBI | -0.0094 (-0.0189; 2e-04) |
|  |  | ≥2 TBIs | -0.0036 (-0.0163; 0.0092) |
| Processing speed | TBI (ref = no TBI) | 1 TBI | -0.0138 (-0.1468; 0.1191) |
|  |  | ≥2 TBIs | -0.0954 (-0.2746; 0.0838) |
|  | Time |  | -0.0929 (-0.0979; -0.0879) |
|  | TBI by time (ref = no TBI) | 1 TBI | 1e-04 (-0.0102; 0.0104) |
|  |  | ≥2 TBIs | 0.0041 (-0.0097; 0.0178) |

*Note*: Beta (β) and 95% confidence intervals (CI) are derived from linear mixed-effects models that included random intercepts and family-relatedness a random effect to adjust for correlation between twin pairs. Time is defined as years from baseline. Models include fixed effects of TBI, time, and a TBI by time interaction term, and are adjusted for baseline age (centered at 57.86 years, the average age of entry into VETSA), race/ethnicity, education, annual family income, young adult cognitive ability (AFQT at age 20) and APOE ε4 carrier status as well as time-varying BMI (standardized), smoking status, alcohol use, substance abuse, relationship status, participation in religious activities, number of close friends, social isolation, and elevated psychiatric symptoms.
