# Supplementary material for: Association Between Traumatic Brain Injury and Cognitive Decline Among Middle-to-Older Aged Men in the Vietnam Era Twin Study of Aging
Source: Neurotrauma Rep. 2024 Jun 17;5(1):563–73. doi: 10.1089/neur.2024.0034 (PMC11257108; doi:10.1089/neur.2024.0034)
Supplement: Supplementary Table S6 [file neur.2024.0034_supplementarytable6.docx]

| **Supplementary Table 6:** Association of any traumatic brain injury with cognitive performance trajectories by APOE ε4 carrier status (excluding ε2/ε4 from ε4 carrier group) over a 12 year of follow up | | | | | |
| --- | --- | --- | --- | --- | --- |
|  |  |  | APOE ε4 carrier status | |  |
|  |  |  | No (n=948) | Yes (n=344) |  |
| Outcome | Model | Term | β (95% CI) | β (95% CI) | *P*_Interaction_ |
| Episodic memory | 1 | TBI | -0.027 (-0.1573; 0.1034) | 0.1634 (-0.0513; 0.3782) | 0.2952 |
|  |  | Time | -0.0442 (-0.0499; -0.0385) | -0.0483 (-0.0574; -0.0391) |  |
|  |  | TBI by time | -0.0021 (-0.0124; 0.0082) | -0.0127 (-0.0292; 0.0037) |  |
|  | 2 | TBI | -0.0047 (-0.1371; 0.1277) | 0.184 (-0.0324; 0.4003) |  |
|  |  | Time | -0.0427 (-0.0487; -0.0366) | -0.0457 (-0.0557; -0.0357) |  |
|  |  | TBI by time | -0.0041 (-0.0147; 0.0065) | -0.0151 (-0.0326; 0.0024) |  |
| Executive function | 1 | TBI | 0.0547 (-0.0654; 0.1748) | 0.1172 (-0.0851; 0.3194) | 0.0125 |
|  |  | Time | -0.065 (-0.0704; -0.0597) | -0.0729 (-0.0814; -0.0644) |  |
|  |  | TBI by time | -0.0026 (-0.0123; 0.007) | -0.0188 (-0.0341; -0.0035) |  |
|  | 2 | TBI | 0.0679 (-0.0531; 0.1889) | 0.1333 (-0.078; 0.3446) |  |
|  |  | Time | -0.0641 (-0.0697; -0.0586) | -0.0706 (-0.0799; -0.0612) |  |
|  |  | TBI by time | -0.0031 (-0.0128; 0.0067) | -0.0205 (-0.0368; -0.0042) |  |
| Processing speed | 1 | TBI | -0.0537 (-0.1919; 0.0845) | 0.0505 (-0.184; 0.285) | 0.1850 |
|  |  | Time | -0.0964 (-0.1021; -0.0907) | -0.0873 (-0.096; -0.0787) |  |
|  |  | TBI by time | 0.0031 (-0.0071; 0.0133) | 0.0017 (-0.0139; 0.0172) |  |
|  | 2 | TBI | -0.0312 (-0.1697; 0.1074) | 0.0446 (-0.1771; 0.2662) |  |
|  |  | Time | -0.0945 (-0.1005; -0.0884) | -0.0846 (-0.0939; -0.0753) |  |
|  |  | TBI by time | 0.0018 (-0.0089; 0.0125) | 5e-04 (-0.0156; 0.0167) |  |
| Note: Beta (β) and 95% confidence intervals (CI) are derived from linear mixed-effects models that included random intercepts and family-relatedness a random effect to adjust for correlation between twin pairs. Time is defined as years from baseline. Model 1 fixed effects of TBI, time, and a TBI by time interaction term, and adjusted for baseline age (centered at 57.86 years, the average age of entry into VETSA), race/ethnicity, education, annual family income, and young adult cognitive ability (AFQT at age 20). Model 2 additionally adjusted for time-varying BMI (standardized), smoking status, alcohol use, and substance abuse, relationship status, participation in religious activities, number of close friends, social isolation, and elevated psychiatric symptoms. Model 3 additionally adjusted for APOE ε4 carrier status. P values for interaction (*P*_Interaction_) were calculated using likelihood ratio tests to compare fully adjusted models with and without a 3-way interaction of APOE ε4 carrier status by TBI by time. | | | | | |
