# Supplementary material for: Association Between Traumatic Brain Injury and Cognitive Decline Among Middle-to-Older Aged Men in the Vietnam Era Twin Study of Aging
Source: Neurotrauma Rep. 2024 Jun 17;5(1):563–73. doi: 10.1089/neur.2024.0034 (PMC11257108; doi:10.1089/neur.2024.0034)
Supplement: Supplementary Table S7 [file neur.2024.0034_supplementarytable7.docx]

| **Supplementary Table 7:** Secondary outcomes for the association of any traumatic brain injury with cognitive performance trajectories over a 12 year of follow up | | | |
| --- | --- | --- | --- |
| Outcome | Model | Term | β (95% CI) |
| Working memory | 1 | TBI | 0.0116 (-0.0859; 0.1091) |
|  |  | Time | -0.0346 (-0.0386; -0.0306) |
|  |  | TBI by time | -0.0047 (-0.0119; 0.0025) |
|  | 2 | TBI | 0.0289 (-0.0702; 0.1279) |
|  |  | Time | -0.0338 (-0.0381; -0.0296) |
|  |  | TBI by time | -0.0053 (-0.0128; 0.0022) |
|  | 3 | TBI | 0.0295 (-0.0695; 0.1285) |
|  |  | Time | -0.0338 (-0.038; -0.0296) |
|  |  | TBI by time | -0.0053 (-0.0128; 0.0022) |
| Verbal fluency | 1 | TBI | -0.0021 (-0.1123; 0.1081) |
|  |  | Time | -0.0253 (-0.0296; -0.0209) |
|  |  | TBI by time | -0.0055 (-0.0133; 0.0023) |
|  | 2 | TBI | 0.0162 (-0.0947; 0.1272) |
|  |  | Time | -0.0243 (-0.0289; -0.0197) |
|  |  | TBI by time | -0.0076 (-0.0157; 6e-04) |
|  | 3 | TBI | 0.0136 (-0.0977; 0.1249) |
|  |  | Time | -0.0245 (-0.0291; -0.0199) |
|  |  | TBI by time | -0.0074 (-0.0156; 7e-04) |
| Semantic fluency | 1 | TBI | 0.053 (-0.0603; 0.1664) |
|  |  | Time | -0.031 (-0.0364; -0.0255) |
|  |  | TBI by time | -0.0079 (-0.0177; 0.002) |
|  | 2 | TBI | 0.0734 (-0.0409; 0.1876) |
|  |  | Time | -0.0302 (-0.036; -0.0244) |
|  |  | TBI by time | -0.0102 (-0.0204; 1e-04) |
|  | 3 | TBI | 0.0755 (-0.0383; 0.1893) |
|  |  | Time | -0.0302 (-0.036; -0.0245) |
|  |  | TBI by time | -0.0102 (-0.0204; 1e-04) |

*Note*: Beta (β) and 95% confidence intervals (CI) are derived from linear mixed-effects models that included random intercepts and family-relatedness a random effect to adjust for correlation between twin pairs. Time is defined as years from baseline. Model 1 fixed effects of TBI, time, and a TBI by time interaction term, and adjusted for baseline age (centered at 57.86 years, the average age of entry into VETSA), race/ethnicity, education, annual family income, and young adult cognitive ability (AFQT at age 20). Model 2 additionally adjusted for time-varying BMI (standardized), smoking status, alcohol use, substance abuse, relationship status, participation in religious activities, number of close friends, social isolation, and elevated psychiatric symptoms. Model 3 additionally adjusted for APOE ε4 carrier status.
