# Supplementary material for: Association Between Traumatic Brain Injury and Cognitive Decline Among Middle-to-Older Aged Men in the Vietnam Era Twin Study of Aging
Source: Neurotrauma Rep. 2024 Jun 17;5(1):563–73. doi: 10.1089/neur.2024.0034 (PMC11257108; doi:10.1089/neur.2024.0034)
Supplement: Supplementary Table S8 [file neur.2024.0034_supplementarytable8.docx]

| **Supplementary Table 8:** Secondary outcomes for the association of any traumatic brain injury with cognitive performance trajectories by APOE ε4 carrier status over a 12 year follow up | | | | | |
| --- | --- | --- | --- | --- | --- |
|  |  |  | APOE ε4 carrier status | |  |
|  |  |  | No (n=948) | Yes (n=394) |  |
| Outcome | Model | Term | β (95% CI) | β (95% CI) | *P*_Interaction_ |
| Working memory | 1 | TBI | 0.0398 (-0.0758; 0.1553) | -0.0108 (-0.1933; 0.1717) | 0.13 |
|  |  | Time | -0.0351 (-0.0399; -0.0303) | -0.0332 (-0.0404; -0.026) |  |
|  |  | TBI by time | -5e-04 (-0.0091; 0.0081) | -0.015 (-0.0281; -0.0019) |  |
|  | 2 | TBI | 0.0519 (-0.065; 0.1688) | 0.0194 (-0.171; 0.2097) |  |
|  |  | Time | -0.0343 (-0.0394; -0.0292) | -0.0329 (-0.0406; -0.0251) |  |
|  |  | TBI by time | -0.001 (-0.0099; 0.008) | -0.0154 (-0.0292; -0.0016) |  |
| Verbal fluency | 1 | TBI | 0.0576 (-0.0747; 0.19) | -0.1429 (-0.3414; 0.0555) | 0.13 |
|  |  | Time | -0.0241 (-0.0294; -0.0188) | -0.0287 (-0.036; -0.0214) |  |
|  |  | TBI by time | -0.0087 (-0.0183; 8e-04) | 0.0027 (-0.0106; 0.016) |  |
|  | 2 | TBI | 0.0834 (-0.0495; 0.2163) | -0.1565 (-0.3604; 0.0473) |  |
|  |  | Time | -0.022 (-0.0276; -0.0163) | -0.0304 (-0.0384; -0.0223) |  |
|  |  | TBI by time | -0.0123 (-0.0223; -0.0024) | 0.005 (-0.0093; 0.0193) |  |
| Semantic fluency | 1 | TBI | 0.0911 (-0.0442; 0.2264) | -0.0744 (-0.2809; 0.1322) | 0.57 |
|  |  | Time | -0.0314 (-0.038; -0.0248) | -0.0297 (-0.0395; -0.0198) |  |
|  |  | TBI by time | -0.0105 (-0.0223; 0.0014) | -0.0021 (-0.0201; 0.016) |  |
|  | 2 | TBI | 0.1136 (-0.0225; 0.2496) | -0.0889 (-0.3019; 0.1241) |  |
|  |  | Time | -0.0305 (-0.0374; -0.0236) | -0.0289 (-0.0398; -0.018) |  |
|  |  | TBI by time | -0.0125 (-0.0246; -4e-04) | -0.0035 (-0.0229; 0.0159) |  |
| *Note*: Beta (β) and 95% confidence intervals (CI) are derived from linear mixed-effects models that included random intercepts and family-relatedness a random effect to adjust for correlation between twin pairs. Time is defined as years from baseline. Model 1 fixed effects of TBI, time, and a TBI by time interaction term, and adjusted for baseline age (centered at 57.86 years, the average age of entry into VETSA), race/ethnicity, education, annual family income, and young adult cognitive ability (AFQT at age 20). Model 2 additionally adjusted for time-varying BMI (standardized), smoking status, alcohol use, substance abuse, relationship status, participation in religious activities, number of close friends, social isolation, and elevated psychiatric symptoms. *P* values for interaction (*P*_Interaction_) were calculated using likelihood ratio tests to compare fully adjusted models with and without a 3-way interaction of APOE ε4 carrier status by TBI by time. | | | | | |
